# Getting bacterial cells into shape

**DOI:** 10.7554/eLife.93719

**Published:** 2023-12-13

**Authors:** Mrinmayee Bapat, Vani Pande, Pananghat Gayathri

**Affiliations:** 1 Biology Division, Indian Institute of Science Education and Research Pune India

**Keywords:** actin-like MreB, polymerization, Geobacillus stearothermophilus, crystal structure, ATPase, electron microscopy, Other

## Abstract

The conformational state of a structural protein in bacteria can vary, depending on the concentration level of potassium ions or the nucleotide bound to it.

**Related research article** Mao W, Renner LD, Cornilleau C, Li de la Sierra-Gallay I, Afensiss S, Benlamara S, Ah-Seng Y, Van Tilbeurgh H, Nessler S, Bertin A, Chastanet A, Carballido-Lopez R. 2023. On the role of nucleotides and lipids in the polymerization of the actin homolog MreB from a Gram-positive bacterium. *eLife* **12**:e84505. doi: 10.7554/eLife.84505.

Many rod-shaped bacteria rely on a protein known as MreB to acquire and maintain their characteristic three-dimensional structure (Jones et al., 2001; van den Ent et al., 2001; Domínguez-Escobar et al., 2011; Garner et al., 2011; van Teeffelen et al., 2011). Like its eukaryotic homolog, the actin protein, MreB filaments are formed of small units that polymerise into two paired strands. The resulting filaments interact with the molecular machinery responsible for building the cell wall of a bacterium, which is mediated through an interaction with the cell membrane. This helps to orient them so that wall synthesis is done in a directed manner. Much like a potter’s hand guides the clay into a cylinder at the wheel, MreB’s circumferential movements in a direction perpendicular to the long axis of the cell help to create a rod-like shape.

It is known that its eukaryotic cousin actin has a pair of dynamic protofilaments, which constantly add and lose monomers. The two ends of the protofilament are different, with a plus and a minus end. The two protofilaments wrap around each other in a parallel manner – the plus goes with plus, and the minus goes with the minus, so the resulting filament is also polar. The plus end grows – i.e., adds new monomers of actin – much faster than the minus end. Newly added actin monomers are bound to the nucleotide ATP (adenosine triphosphate). When they get incorporated into a filament, the bound ATP loses a phosphate and becomes ADP (adenosine di-phosphate) through a chemical reaction called hydrolysis. When the filament depolymerises from the minus end, the ADP-bound monomers are released, and ADP is replaced by ATP: the ATP-bound monomers are ready to be reincorporated into the filament at the plus end. This process is also known as treadmilling, as the molecules lost at one end get reincorporated at the other end.

All MreB filaments characterised to date have antiparallel protofilaments and therefore are not polar. As a consequence, these filaments cannot exhibit the same treadmilling-like dynamics found in actin. Moreover, the ability to bind and hydrolyse ATP also varies between bacterial species (Bean and Amann, 2008; Esue et al., 2005; Nurse and Marians, 2013; Pande et al., 2022). While residues for ATP-binding are conserved in MreBs, and MreBs have been shown to be able to bind and hydrolyse ATP, it is still unclear what role ATP plays in the assembly, disassembly and movement of MreB filaments (Bork et al., 1992).

Now, in eLife, Rut Carballido-López and colleagues at research institutes in France and Germany – including Wei Mao (Université Paris-Saclay) as first author – report new insights into the dynamics of MreB in the bacterium *Geobacillus stearothermophilus* (Mao et al., 2023). Mao et al. used a combination of techniques to study the structure of MreB filaments and how they assemble and disassemble. They used liposomes to mimic the cell membrane of a bacterium and to function as a scaffold that facilitates the polymerisation of GsMreB (Hussain et al., 2018; Pande et al., 2022; Salje et al., 2011; van den Ent et al., 2014).

First, using electron microscopy, the researchers studied the effect of lipids on the assembly of the protein filaments. This revealed that polymers could only form in the presence of lipids, which suggests that the filaments assemble on the cytoplasmic side of the cell membrane. Moreover, the process was dependent on ATP but not ADP or AMPPNP, an analog of ATP that cannot be hydrolysed to ADP and remains frozen in the ATP state. This is in contrast to another bacterium called *Spiroplasma*, in which MreB5 had been shown to polymerise independently of ATP and lipids (Pande et al., 2022; Takahashi et al., 2022).

To better understand the dynamics underlying the protein assembly, Mao et al. quantified the amount of protein bound to the lipids in the presence of ATP and measured the rate of ATP hydrolysis. The experiments revealed that as ATP gets hydrolysed, the protein filaments also disassemble, supporting the existence of an ATP-hydrolysis dependent dynamics in MreB filaments.

In the absence of a polarity, MreB filaments can grow or shrink equally from either end, though this has not been measured. However, the antiparallel arrangement of MreB filaments exposes a surface on the filament that can bind to the cell membrane, which has also been observed in MreBs of other bacterial species (Salje et al., 2011).

Positively charged ions are known to further influence polymerization of actin (Tellam, 1985). Mao et al. used cryo-electron microscopy to find out if different concentrations of potassium change the properties of the filaments and consequently the shape of the liposome (Figure 1). Under low potassium concentrations (100 mM), MreB filaments appear curved and flexible, and created indents into the smooth spherical surface of the liposome (Figure 1A and B). Under high concentrations (500 mM), the filaments were long, straight and stiff and elongated the liposome (Figure 1C).

**Figure 1.**
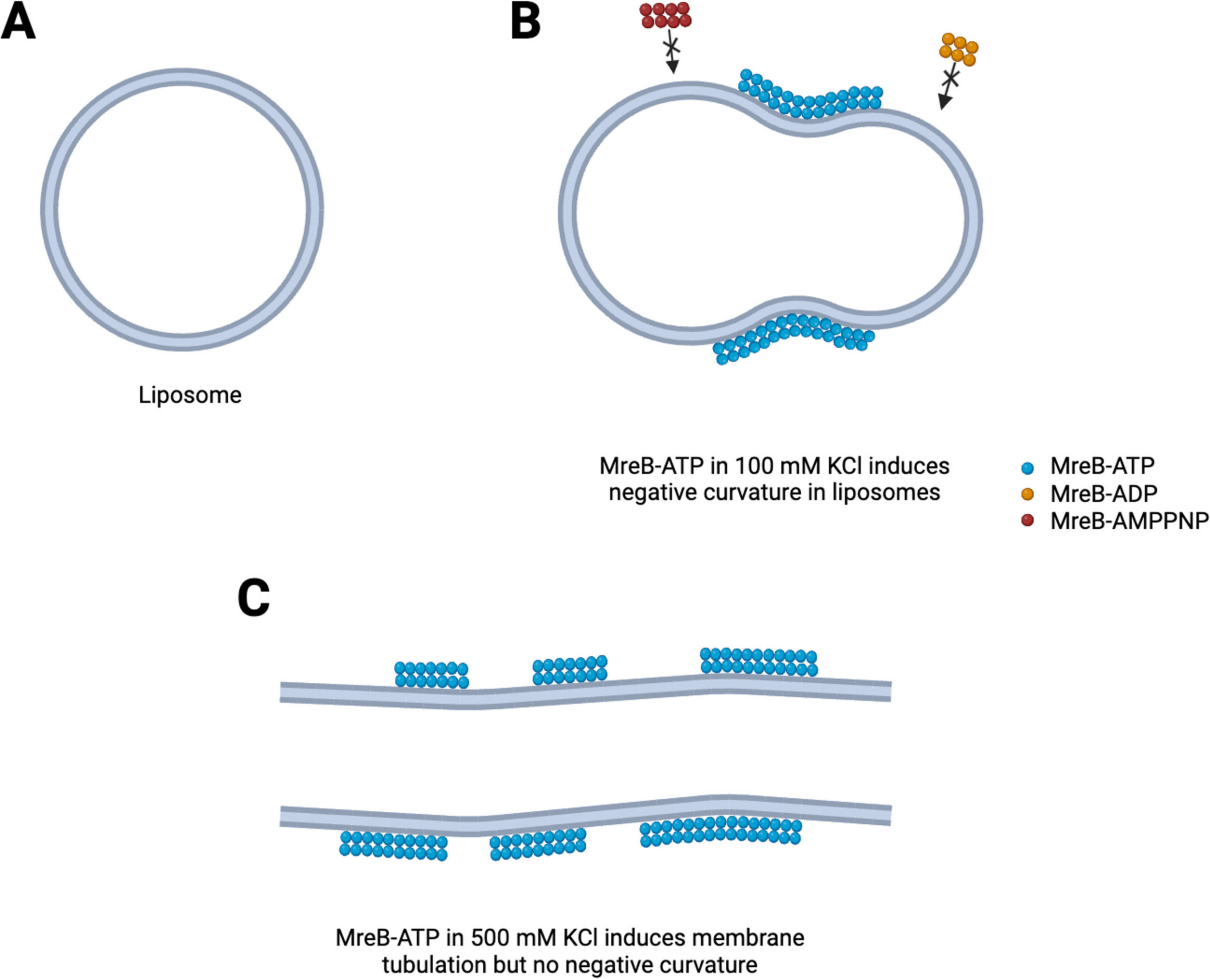
Mechanisms to shape bacterial cells. (**A**) In the absence of the protein MreB, cell-like structures called liposomes are spherical. (**B**) In the presence of ATP (blue circles) but not ADP or AMPPNP (red and orange circles), MreB filaments induce a negative membrane curvature on liposomes when salt concentrations are low. (**C**) When salt concentrations are high, MreB filaments induce a tubular shape in the liposome.

Image credits: Figure created using Biorender.

These observations demonstrate that the conformational state of the MreB filaments can vary, based either on the nature of the nucleotide-bound state or the level of potassium ions. A bent conformation of MreB filament could possibly match the curvature of the liposome (resulting in a curvature-sensing role of the filaments), while a rigid or stiff filament could chisel the liposome into a different shape (membrane remodelling). This might help the filament to identify regions of the membrane that has to be ‘ironed out’ and then move to the next area to be ‘straightened’.

More research is needed to elucidate the features or states that differentiate between curvature sensing and membrane remodelling. A better understanding of the interplay between nucleotide binding, filament assembly and membrane binding will help researchers to identify the mechanism of circumferential movement that provides rod-shaped bacteria with their unique look.
